# Janus Nanostructures from ABC/B Triblock Terpolymer Blends

**DOI:** 10.3390/polym11071107

**Published:** 2019-06-30

**Authors:** Andrea Steinhaus, Deepika Srivastva, Arash Nikoubashman, André H. Gröschel

**Affiliations:** 1 Physical Chemistry and Center for Nanointegration Duisburg-Essen (CENIDE), University Duisburg-Essen, 47057 Duisburg, Germany; 2 Institute of Physics, Johannes Gutenberg University Mainz, Staudingerweg 7, 55128 Mainz, Germany

**Keywords:** block copolymers, bulk morphologies, DPD simulations, Janus nanostructures, polymer blending

## Abstract

Lamella-forming ABC triblock terpolymers are convenient building blocks for the synthesis of soft Janus nanoparticles (JNPs) by crosslinking the B domain that is “sandwiched” between A and C lamellae. Despite thorough synthetic variation of the B fraction to control the geometry of the sandwiched microphase, so far only Janus spheres, cylinders, and sheets have been obtained. In this combined theoretical and experimental work, we show that the blending of polybutadiene homopolymer (hPB) into lamella morphologies of polystyrene-*block*-polybutadiene-*block*-polymethylmethacrylate (SBM) triblock terpolymers allows the continuous tuning of the polybutadiene (PB) microphase. We systematically vary the volume fraction of hPB in the system, and we find in both experiments and simulations morphological transitions from PB-cylinders to perforated PB-lamellae and further to continuous PB-lamellae. Our simulations show that the hPB is distributed homogeneously in the PB microdomains. Through crosslinking of the PB domain and redispersion in a common solvent for all blocks, we separate the bulk morphologies into Janus cylinders, perforated Janus sheets, and Janus sheets. These studies suggest that more complex Janus nanostructures could be generated from ABC triblock terpolymers than previously expected.

## 1. Introduction

Block copolymers (BCPs) consist of two or more incompatible segments that microphase separate on a characteristic length scale of 10–100 nm [1]. Their ability to spontaneously form nanostructures of defined geometry is extensively used in soft matter and materials science to create functional materials in dilute solution, in emulsions, and in bulk [2]. Bulk morphologies of AB diblock copolymers have been particularly well studied and applied as templates for inorganic materials [3], for catalysis or energy conversion [4,5], as nanoporous membranes [6,7], lithographic masks [8,9], and in photonics [10]. By synthetic variation of the volume fractions through block lengths *ϕ*_A_ (*ϕ*_B_ = 1 − *ϕ*_A_), the geometry of the nanostructure is controlled from spheres to cylinders, as well as gyroid and lamella morphologies. The gyroid morphology is attractive for high surface area applications [3], however, it is only found in a rather narrow window of the microphase diagram, i.e., as a transition structure between cylinders and lamellae. Depending on the chemical nature of the monomers, it can be challenging to target specific microphases synthetically. The choice of solvent, evaporation rate, and external fields are suitable means to manipulate the resulting morphology [11,12].

ABC triblock terpolymers, i.e., BCPs with three different blocks, naturally have a larger number of interaction parameters and thus display a much more complex microphase behavior as confirmed by theory and experiment [13,14,15,16,17]. Pioneered by Reimund Stadler already in the early 1990s [18,19,20,21,22,23], many research groups have since then invested efforts to analyze and understand the microphase behavior of ABC triblock terpolymers [24,25,26,27,28]. Lamellar morphologies are of particular interest as they give access to Janus nanoparticles (JNPs) by crosslinking of the “sandwiched” B microphase. Depending on the weight fraction of the B block, *f*_B_, Janus spheres, cylinders and sheets/discs have been synthesized [29,30,31,32]. Such polymeric JNPs with one dimension in the range of 10–100 nm are attractive colloidal surfactants [33] and useful for applications related to interfacial stabilization [34,35,36,37]. Although the desorption energy of JNPs from oil-water interfaces scales with particle radius [38], JNPs with cores of only 10 nm radius reduce interfacial tension considerably and are able to cause interfacial jamming [39,40,41,42].

Given the complexity of the ternary phase diagram, it is likely that more Janus geometries still remain undiscovered. However, synthetic variation of block weight fractions and/or monomer chemistry requires considerable experimental effort making the exploration of suitable morphologies challenging. In that regard, blending of low molecular weight additives is a convenient tool to alter domain volumes of ABC triblock terpolymers in a continuous manner [43]. Supramolecular complexation with small molecular weight hydrogen bond donors is one possibility to induce gradual changes to the morphology if hydrogen bond acceptors are present in the polymer [44]. For instance, modification of the middle block of polystyrene-*block*-poly(4-vinylpyridine)-*block*-poly(*tert*-butyl methacrylate) with azobenzene derivatives induced a morphological transition from P4VP cylinders to a perforated P4VP/Azo lamella [45]. Homopolymers were also used to swell individual phases and invoke morphological transition [46,47,48], while compatible diblock copolymers or triblock terpolymers were blended to other triblock terpolymers to explore the formation of novel superlattices [49,50,51,52,53,54,55]. These examples encourage the idea that any “sandwiched” microphases could be altered by swelling with a compatible low molecular weight homopolymer to change the geometry of the resulting JNPs.

In a previous work, we studied the self-assembly of polystyrene-*block*-polybutadiene-*block*-polymethylmethacrylate (SBM) triblock terpolymers in the confinement of microemulsions and obtained different JNP morphologies from the same SBM terpolymer [56]. These results suggest that an *f*_PB_ of 22 wt% should be close to a morphological transition, which makes this candidate ideal for studying the effect of polybutadiene homopolymer (hPB) blending on the bulk morphology. In this contribution, we demonstrate that blending of specific amounts of hPB allows the continuous tuning of lamellar bulk morphologies of SBM from polybutadiene (PB) cylinders to a continuous PB lamella. Here, we first observe lateral fusion of the PB cylinders into hexagonally perforated PB lamellae, before perforations are closed upon further addition of hPB. Complementary dissipative particle dynamics (DPD) simulations confirm these structural transitions at similar amounts of added hPB. Further, the simulations reveal that the added hPB is dispersed homogeneously throughout the PB microdomains. Crosslinking of the PB domains and redispersion of the bulk film in tetrahydrofuran (THF) results in JNPs where perforated Janus nanosheets pose interesting materials in terms of directional adsorption to interfaces and for separation applications [57].

## 2. Materials and Methods

### 2.1. Materials

Chloroform was received from VWR in p.a. quality and used as such. Polybutadiene homopolymer (hPB, *M*_n_ ≈ 2000 g∙mol^−1^ and *Đ* = 1.39) and sulphur monochloride (98%) were obtained from Sigma Aldrich. Osmium tetroxide (4% aqueous solution) was obtained from Science Services (Munich, Germany). The S_40_B_22_M_38_ triblock terpolymer with *M*_n_ = 143,000 g·mol^−1^ and *Đ* = 1.05 was synthesized as described elsewhere [18,19,20,21,22,23]. For SEC traces see Figure S1 of the Supporting Information (SI).

### 2.2. Preparation of SBM Bulk Films

For bulk film preparation, hPB was first dissolved in CHCl_3_ to obtain a solution of 20 g·L^−1^. Then, in a 40 mL glass vial, 50 mg of terpolymer was dissolved in 2 mL CHCl_3_, mixed with an appropriate amount of homopolymer solution according to Table 1 and stirred for 2 h. The solvent was evaporated over the course of 5 days under constant CHCl_3_ atmosphere. After film formation, the vial was cooled with liquid nitrogen and the bulk film released by breaking the vial.

### 2.3. Crosslinking of SBM Bulk Films

The bulk films were placed in a glass chamber and 100 µL S_2_Cl_2_ was added in a separate container, before the chamber was closed. Crosslinking proceeded via gas phase overnight. The next day, the films were removed and left for evaporation of excess sulphur monochloride overnight. For the transmission electron microscopy (TEM) measurements, bulk films were cut into ultrathin sections on a Leica EM UC7 ultramicrotome, deposited on a carbon-coated copper grid (200 mesh, Science Services), and stained with OsO_4_ vapor for 3 h prior to measurements. If redispersion was desired, the bulk films were dissolved in THF to obtain a concentration of 1 g·L^−1^, 3 days prior to measurements at least.

### 2.4. Transmission Electron Microscopy (TEM)

The TEM measurements were performed on a JEM-1400 Plus TEM (JEOL, Freising, Germany), operated at an accelerating voltage of 120 kV, a point resolution of 0.38 nm, as well as a line resolution of 0.2 nm. Images were recorded with a 16-bit 4096 × 4096 pixel CMOS digital camera and processed through the FIJI open-source software package. For sample preparation, one drop of the bulk film solution (*c* = 1 g·L^−1^) was deposited on a carbon-coated copper grid (200 mesh, Science Services). All samples were stained with OsO_4_ for 3 h prior to measurements.

### 2.5. Simulation Model and Method

In our DPD simulations we used a coarse-grained bead-spring model, where each SBM triblock terpolymer was represented by N=40 spherical beads of diameter a and unit mass m, connected through harmonic springs. We matched the weight fraction of the individual blocks to the experiments, leading to $N_{S}=16$, $N_{B}=8$, and $N_{M}=16$ beads for the S, B, and M blocks, respectively. Using this level of coarse-graining, each bead had a diameter of a≈2.2 nm and contained between 35 and 60 monomers depending on the particle type. Thus, each bead effectively represented a coil-like segment of the terpolymer. The effective pair interaction between such coils is rather soft and bounded [58,59], and therefore we used the typical soft repulsion for the conservative forces, $F_{C}$, between bonded and nonbonded monomers [60]

(1) $$F_{C}\left(r\right)=\{\begin{array}{cc} A_{ij}\left(1-r/a\right)\hat{r}, & r<a\\ 0, & r\geq a \end{array}$$

where, r is the radial distance between the center of two particles and $\hat{r}$ is the unit vector connecting them. The parameter $A_{ij}$ controls the maximum repulsion between particles of types i and j (the specific values for $A_{ij}$ are discussed further below and summarized in Table 2. Bonds within a chain are modelled using harmonic springs with force

(2) $$F_{C}^{B}\left(r\right)=-kr$$

with spring constant set to k=4 in the reduced simulation units [61].

In addition to these conservative forces, the particles are also subject to pairwise dissipative, $F_{D}$, and random forces, $F_{R}$, which impart thermal fluctuations and drag while also serving as a thermostat on the DPD particles. The terms are given by

(3) $$F_{D}\left(r\right)=-\gamma_{ij}w\left(r\right)\left(\hat{r}\cdot \Delta v\right)\hat{r}$$

(4) $$F_{R}\left(r\right)=\sqrt{\gamma_{ij}w\left(r\right)}\xi \hat{r}$$

where, $\gamma_{ij}$ is the drag coefficient between particles of type i and j, w(r) is a weight function, and Δv is the velocity difference of the particle pairs. The variable ξ is an independent random variable drawn for each particle pair that obeys ξ(t)=0 and $\xi \left(t\right)\xi \left(t{}^{\prime }\right)=2k_{B}T\delta \left(t-t^{\prime }\right)$ to satisfy the fluctuation-dissipation theorem [62]. In this work, we used the same drag coefficient $\gamma_{ij}=\gamma =4.5$ independent of particle type. For the weight function, we employed the (standard) form

(5) $$w\left(r\right)=\{\begin{array}{cc} {\left(1-r/a\right)}^{2}, & r<a\\ 0, & r\geq a \end{array}$$

We chose a particle number density of $\rho =3a^{-3}$ and set the interaction strength between particles of the same type to $A_{ii}=25$. Note, that this parameter combination has been originally determined by Groot and Warren to match the compressibility of water [60], but since then this choice has been widely used to model other (incompressible) liquids and polymer melts [61,63,64,65]. Groot and Warren have also derived a relationship between $A_{ij}$ and the Flory–Huggins χ parameter [60]

(6) $$A_{ij}=A_{ii}+3.497\chi$$

In Section 3, we discuss how the specific χ values have been obtained. The resulting values for $A_{ij}$ are listed in Table 2 below. Simulations were conducted in a rectangular box with periodic boundary conditions in all three Cartesian directions. The box dimensions were chosen as $L_{x}=40a$ and $L_{y}=L_{z}=\sqrt{3}L_{x}/2$, so that a hexagonal arrangement of cylinders fit into the box. For the systems containing homopolymers, the box volume was adjusted accordingly to maintain a particle number density of $\rho =3a^{-3}$. All polymers were initially placed randomly at positions in the box and the cross interaction was set to $A_{ij}=A_{ii}$ (i.e., χ=0). Then, $A_{ij}$ was linearly increased to its final value over $5\times 10^{6}$ steps with a time step Δt=0.05. We performed three independent simulations per state point to gather statistics.

## 3. Results and Discussions

**Scheme 1:**
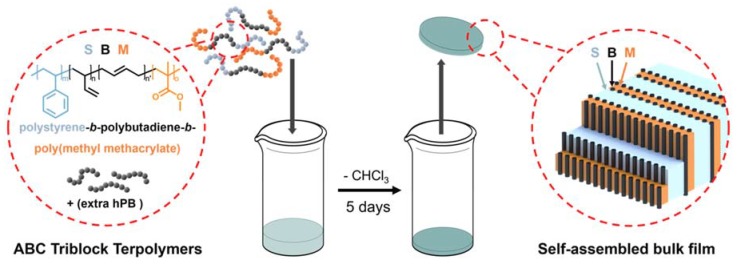
Preparation of SBM/B bulk films. Chemical structure of SBM, casting of the bulk film and lamellar PS/PMMA morphology and varying PB domain depending on polybutadiene homopolymer (hPB) content.

The preparation of bulk films from polystyrene-*block*-polybutadiene-*block*-polymethylmethacrylate/polybutadiene (SBM/B) blends is outlined in Scheme 1 and preparative details are found in the Materials and Methods section. At first, the SBM triblock terpolymer is dissolved in 1 mL CHCl_3_ (*c*_SBM_ = 50 g∙L^−1^), mixed with a suitable amount of hPB solution (*c*_hPB_ = 20 g∙L^−1^), and stirred for 3 h to ensure complete mixing of both components. The SBM terpolymer in this study has a weight fraction of *f*_PB_ = 22 wt% and equally-sized outer blocks of PS and PMMA. The amount of added hPB in relation to PB and the resulting weight fractions are summarized in Table 1. Evaporation proceeded in a 40 mL glass vial over the course of five days under constant CHCl_3_ atmosphere. Upon slow evaporation of CHCl_3_, the SBM/B blend dried into a solid bulk film, where polymer chains microphase separated into distinct microdomains. Irrespective of the weight fraction, the PB microdomain always remains sandwiched between PS and PMMA lamellae. The bulk film was removed by freezing in liquid nitrogen and breaking the glass vial. Selective crosslinking of PB with sulphur monochloride (S_2_Cl_2_) vapor fixates the morphology and allows its redispersion in THF. Since PS and PMMA are fully soluble in THF, the bulk film separates into nanoparticles with a crosslinked PB core.

By mixing hPB into the SBM morphology, we increased the volume of the PB microphase (Table 1) and gradually changed its geometry. The triblock terpolymer SBM-0 had a molecular weight of 143,000 g∙mol^−1^ with *Đ* = 1.05 to ensure sufficiently strong microphase separation. The molecular weight of hPB was approximately *M*_n_ ≈ 2000 g∙mol^−1^ to ensure its complete incorporation. The amount of added hPB was calculated relative to the PB microdomain in the SBM terpolymer, i.e., 100% hPB (SBM-100) equals twice the amount of PB in pure SBM-0. As *f*_PB_ increases, the composition of the terpolymer changes, resulting in equal-sized polymer blocks at 75% hPB (SBM-75). Previous investigations on bulk film structures of SBM resulted in lamellar microphases for similar compositions [20].

**Figure 1:**
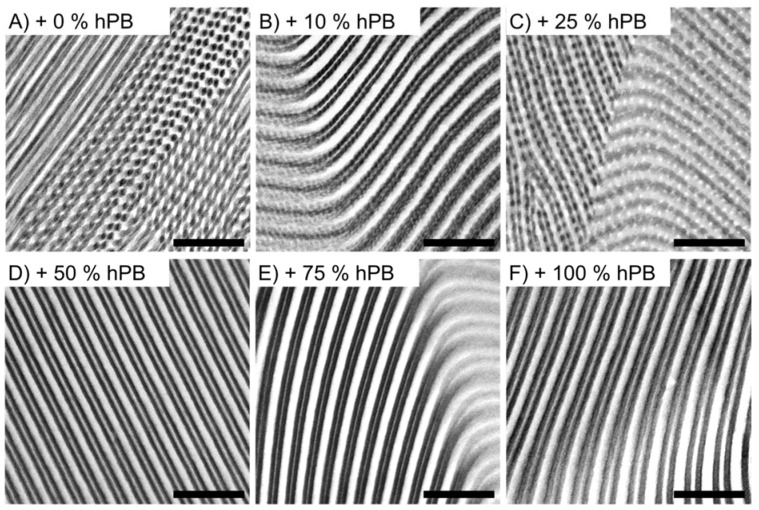
Transmission electron microscopy (TEM) images of bulk morphologies of SBM/B with varying weight fractions of added hPB. TEM images of resulting bulk film from 0% (**A**) to 100% (**F**) hPB in relation to PB domain in SBM. (All samples were stained with OsO_4_ to enhance the contrast of PB, scale bars are 200 nm).

To obtain information about the microphase behavior, the bulk films were cut into ultrathin sections and analyzed by TEM imaging. Before cutting, the PB microphases were crosslinked with S_2_Cl_2_ vapor to enhance the film stability. PB is liquid at room temperature, which softens the film causing problems when cutting, especially with an increasing amount of added hPB. The TEM images of SBM morphologies are summarized in Figure 1, ranging from 0% (A) to 100% (F) of blended hPB. To enhance their contrast, the films were stained with OsO_4_, which bonds covalently to the PB domains causing them to appear dark. After staining, the following three microphases are clearly distinguished: two lamellar microphases, PS and PMMA, separated by an alternating sequence of PB cylinders in between (Figure 1A). Despite similar weight fractions, the PMMA lamellae appear much thinner as compared with the PS lamellae, because PMMA shrinks under e-beam irradiation due to degradation. The PB cylinders appear as black dots when cut perpendicular to the long axis, whereas, they appear as stripes, when cut alongside the cylinder axis (both features are seen at the grain boundary). In certain areas, we find a second morphology, where the PB microphases merge into a mesh-like network, which is a first indication of a morphological transition. The network microstructure is observed more frequently after adding 10 wt% hPB (Figure 1B, SBM-10). In addition, the hexagonally ordered cylinders merge with each other and could not be clearly distinguished anymore indicating their coalescence. However, the PB microphase is not completely lamellar at this point, as it does not show homogeneous contrast. This trend does become even more evident at 25 wt% hPB (Figure 1C, SBM-25), where the PB microphases appear as dotted lines. The grain boundary gives a view on this morphology from two sides and is reminiscent of a perforated lamella. Perforated core-shell lamellae have been reported before with very similar appearance in TEM [66]. In our case, the perforation is formed by the middle block and is thus sandwiched between adjacent PS/PMMA lamellae. Above 50 wt% hPB exclusively lamella-lamella morphologies was obtained (Figure 1D–F). Overview images of each bulk morphology are given in Figure S2 of the SI.

Naturally, the thickness of PB domains increases with respect to the amount of additional hPB, which was also measured by grey scale analysis as summarized in Table 1 and the SI (Figures S3–S8). The PB thickness increases from approximately 11 nm (SBM-0) to 15 nm (SBM-10) when an amount of hPB up to 10 wt% is added. We attribute this increase in thickness to the swelling of the cylinders with hPB. At 25 wt%, the thickness decreases again to ca. 12.8 nm (SBM-25) because the cylinders merge laterally into the perforated mesh causing redistribution of the cylinder volume across a larger two-dimensional (2D) area between the PS/PMMA lamellae. Further addition of hPB does not seem to have an effect on the thickness that remains approximately the same with 12.6 nm at 50 wt% hPB (SBM-50) and 12.9 nm at 75 wt% hPB (SBM-75). The added hPB does not contribute to the thickness, because it is distributed within the PB microphase, filling the perforations of the 2D perforated lamella leading to a complete PB lamella. After complete filling, the thickness increases again reaching a maximum of 15 nm at 100 wt% hPB (SBM-100). It should be pointed out, that the cutting process always leads to film deformation. Polymer domains, therefore, may appear distorted in some areas, so that high precision in greyscale analysis is demanded. Values of PB thickness might, therefore, not be completely accurate in total, however they are applicable to make statements about morphological changes.

**Scheme 2:**
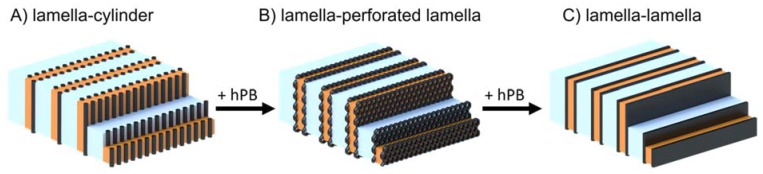
Schematics of SBM/B bulk morphologies with increasing hPB content.

The overall process of PB middle phase transition is outlined schematically in Scheme 2. In general, we start with hexagonally stacked cylinders that are not connected to each other, also known as the lamellar-cylinder morphology. By adding hPB, the cylinders first increase in volume, before they merge into a perforated network, spreading between PS and PMMA layers, i.e., they form a lamella-perforated lamella morphology. This network is then transformed into a continuous PB lamella upon further blending of hPB into the SBM morphology. We believe that the perforated PB lamellae always form between the PS/PMMA lamella and are not connected to adjacent PB layers (e.g., by interpenetrating networks), which is confirmed by our simulations where the PB layers are always well separated from each other (see below). This will be important for the separation of crosslinked layers as discussed later.

**Figure 2:**
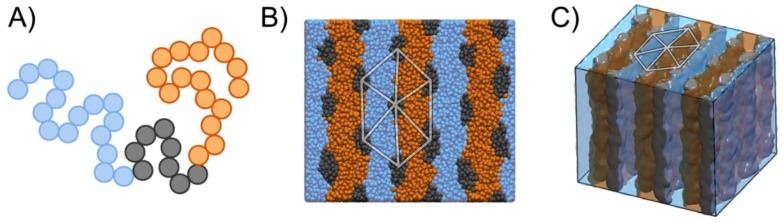
Dissipative particle dynamics (DPD) simulations of an SBM triblock terpolymer melt. (**A**) Schematic representation of the SBM triblock terpolymer used in the DPD simulations. The S, B, and M beads are shown in blue, grey, and orange, respectively. (**B**) Top view showing the lamellar ordering of the PS and PMMA blocks, and the hexagonal arrangement of the cylinders formed by the PB block. (**C**) Three-dimensional view of the simulation box. The white lines in (b) and (c) illustrate the hexagonal ordering of the cylinders. All simulation snapshots have been rendered using visual molecular dynamics [69].

**Figure 3:**
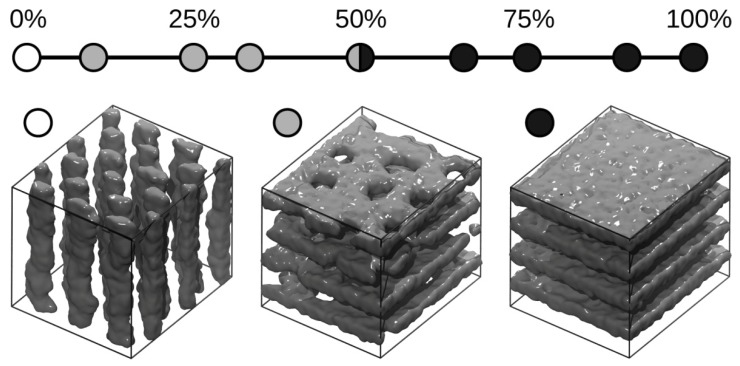
Morphology of the PB microphase as a function of hPB fraction. The PS and PMMA blocks form lamellae in all cases, and they have been omitted for clarity. The symbols indicate results at ϕ=0%, 10%, 25%, 33%, 50%, 66%, 75%, 90%, and 100%. The snapshots show typical configurations of cylinder, perforated lamella, and lamella structures at ϕ=0%, 33%, and 90%, respectively.

The discussed morphological transitions were also found in our DPD simulations, as shown by the simulation snapshots in Figure 2 and Figure 3. In order to replicate accurately the experimental systems, we first determined the Flory–Huggins interaction parameters, χ. For the S–M interactions, we used the expression for χ obtained for SM diblock copolymers via small-angle neutron scattering experiments [67], $\chi_{\mathrm{SM}}=0.028+3.9/T$, leading to a value of $\chi_{\mathrm{SM}}=0.041$ at room temperature (T=298K). For the S-B interactions, we used the expression for χ extracted from the small-angle x-ray scattering analysis of concentration fluctuations in S-B mixtures [68] $\chi_{\mathrm{SB}}=6.96\times 10^{5}/T^{2.65}$, resulting in a value of $\chi_{\mathrm{SB}}=0.19$ at room temperature. We were unable to find literature data for the χ parameter for the B-M interactions, and therefore estimated $\chi_{\mathrm{BM}}$ using the Hildebrand–Scott relation

(7) $$\chi_{ij}\approx {\left(V_{i}V_{j}\right)}^{1/2}{\left(\delta_{i}-\delta_{j}\right)}^{2}/RT$$

Here, $\delta_{i}$ and $\delta_{j}$ are the Hildebrand solubility parameters, $V_{i}$ and $V_{j}$ are the molar volumes of molecules i and j, respectively; and R is the ideal gas constant. Using $\delta_{B}=8.31{\;\mathrm{cal}}^{1/2}{\mathrm{cm}}^{-3/2}$ and $V_{B}=59.7\;{\mathrm{cm}}^{3}/\mathrm{mol}$ for B in combination with $\delta_{M}=9.29{\;\mathrm{cal}}^{1/2}{\mathrm{cm}}^{-3/2}$ and $V_{M}=87.1\;{\mathrm{cm}}^{3}/\mathrm{mol}$ for M, we find $\chi_{\mathrm{BM}}=0.12$. Note that these χ parameters should not be directly used in the simulations, since in our model, multiple monomers are grouped into a single bead. To account for this mapping, we employed a scaled interaction parameter $\chi_{\mathrm{eff}}$ in the simulations to reach the same χN values as in the experiments. Table 2 shows a summary of the interaction parameters.

The molecular weight of the used hPB is approximately *M*_n_ = 2000 g∙mol^−1^, which translates to a chain length of $N_{\mathrm{hPB}}\approx 0.6$, for our level of coarse graining. Such a non-integer value for $N_{\mathrm{hPB}}$ is, however, unphysical, and therefore we used $N_{\mathrm{hPB}}=1$ in our simulations, i.e., each hPB is represented by a single bead. This choice has two consequences: (i) The hPB in the simulations correspond to somewhat longer homopolymers than used in the experiments, and, more importantly; (ii) the simulated hPB lack conformational degrees of freedom. However, given the rather low molecular weight of the hPB as compared with the SBM terpolymer, we expect that the conformational details of the homopolymers are negligible. Figure 2B and C show results for the pure SBM system, where the PS and PMMA blocks self-assemble into lamellae (blue and orange), while the PB blocks form hexagonally arranged cylinders (grey) located at the interfaces between the PS and PMMA domains. We estimate a domain spacing of $D_{L}\approx 29\;\mathrm{nm}$ for the lamella, and $D_{C}\approx 25\;\mathrm{nm}$ for the triangular lattice of the cylinders, which is in almost quantitative agreement with the experimental finding ($D_{L}\approx 32\;\mathrm{nm},\;D_{C}\approx 26\;\mathrm{nm}$). Looking closer at the cross sections of the cylinders, it appears that they are not perfectly circular but somewhat compressed along the direction perpendicular to the lamellae. Such a slight ellipsoidal deformation of the PB cylinder has also been observed in the experiments (cf. Figure 1A), and it might originate from the asymmetry between the S-B and B-M interactions (see Table 2) which leads to asymmetric interfacial tensions.

We next studied the microphase behavior upon adding hPB to the system (Figure 3). Here, we varied the amount of hPB in the melt from ϕ=0% to ϕ=100%, where a value of ϕ=100% indicates that there is the same amount of PB in the system in the form of homopolymer and terpolymer (corresponding to SBM-100). For all investigated cases, the PS and PMMA blocks formed lamellae, but the morphology of the PB blocks changed. For 10% ≤ϕ≤33%, the PB blocks microphase separate into perforated lamellae, which became thicker and less porous with increasing ϕ. At ϕ=50%, we observed a transition from perforated lamellae to lamellae. For the remaining investigated cases, 66% ≤ϕ≤100%, we always found lamellar structures, where the thickness of the PB lamellae increased by roughly 15% with increasing ϕ, as also observed in the experiments (cf. Table 1).

**Figure 4:**
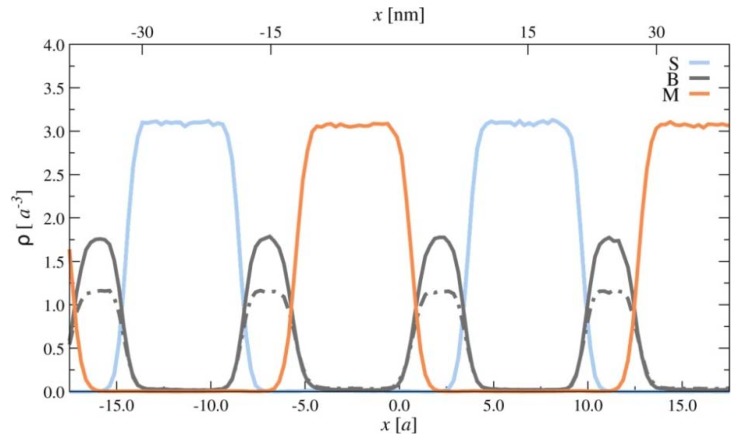
Monomer density distribution of SBM terpolymer (solid lines) with ϕ=75% hPB (dashed line) in the lamellar phase. The x-axis lies perpendicular, relative to the lamellae.

We also investigated the distribution of hPB in the system and found that the homopolymers are uniformly distributed in the PB microphase for all investigated ϕ values. Figure 4 shows, for example, the monomer number density for a lamellar system with ϕ=75% perpendicular to the lamellae, highlighting the clear separation between PS and PMMA microdomains through interstitial PB layers. These PB lamellae consist of PB blocks from SBM terpolymers and hPB, where the local number (or volume) fraction of the two contributions is close to the global fraction, ϕ.

**Figure 5:**
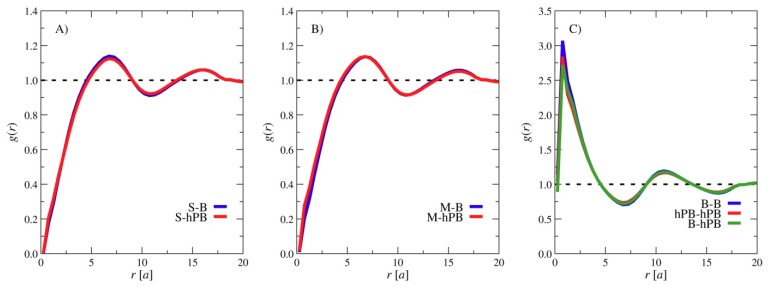
Radial pair distribution function, g(r), between S, B, and M monomers in the system for ϕ=75% hPB.

To further quantify the distribution of hPB in the system, we computed the radial pair distribution function, g(r), for S-B, M-B, and B-B monomer pairs, distinguishing B monomers from terpolymers and homopolymers. For all investigated cases, the radial pair distributions were virtually indistinguishable with respect to the different sources of B monomers in the system, as shown in Figure 5 for the exemplary case ϕ=75%. Further, the g(r) data for the S-B and S-M pairs are very close to each other (Figure 5A,B), which is due to the similar χ values for these two pairs. There are some small, but notable, differences for the B-B data (Figure 5C) at small interparticle distances, r, which originate from the correlations between bonded B monomers in the SBM terpolymers.

**Figure 6:**
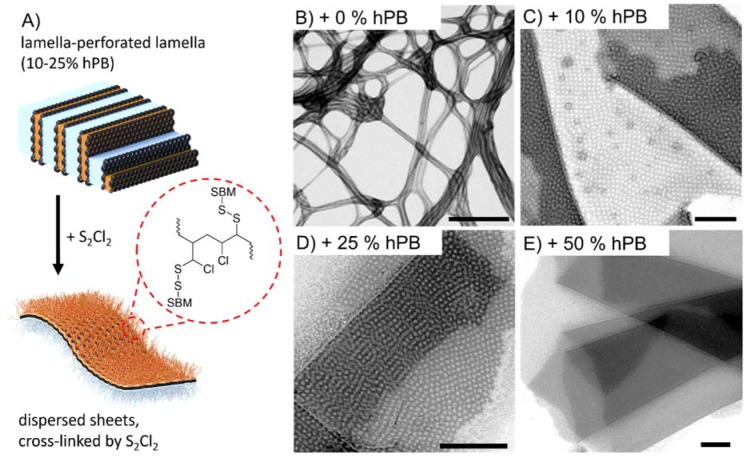
Crosslinking and redispersion of SBM/B morphologies into Janus nanostructures. (**A**) Schematic of lamella-perforated lamella morphology and perforated Janus sheets after crosslinking. (**B–E**) TEM images of crosslinked Janus nanostructures, redispersed in THF.

Finally, we crosslinked the PB domains of the bulk morphologies and redispersed them in a good solvent for all three blocks (THF) to analyze the resulting nanostructures. Crosslinking the PB domain fixes the microdomain geometry, while the PS and PMMA outer chains are covalently anchored to the PB core and stabilize discrete nanostructures when exposed to the solvent. All obtained structures have a Janus character, i.e., PS and PMMA are separated from each other on two distinct sides of the nanoparticles. Figure 6A exemplifies crosslinking and separating of lamella-perforated lamella morphologies into perforated Janus sheets to underline that PS and PMMA polymer chains are connected to the PB network but are completely separated from each other. The TEM results of the nanostructures are summarized in Figure 6B–E. Starting with SBM-0, Figure 6B shows Janus cylinders that originate from the lamella-cylinder morphology. The cylinders are several micrometers in length which underlines the long-range order within the grain. The Janus character is clearly seen from the assembly behavior, i.e., the cylinders align alongside their PS hemisphere as visible from the grey contrast between the dark cylinder cores.

At 10 wt% and 25 wt% we find monolayer sheets with relatively uniform perforations (Figure 6C,D). The pores have an average diameter of *d*_p_ ≈ 20 nm that are separated by 16 ± 4 nm of PB membrane, which corresponds roughly to the diameter of the PB cylinders (supporting the assumption of merging cylinders). Monolayer sheets underline our assumption that the added hPB is distributed between the cylinders and did not penetrate through adjacent PS and PMMA lamellae to form a bicontinuous morphology. Although the sheets are not connected with each other, the sheets overlap on the TEM grid or roll up (darker areas). Similar nanostructures were observed before for terpolymer-based core shell nanosheets of polybutadiene-*block*-poly(2-vinylpyridine)-*block*-poly(*tert*-butyl methacrylate) [66] and blending of hPB into polybutadiene-*block*-poly(*tert*-butyl methacrylate)-*block*-poly(2-(dimethyl-amino)ethyl methacrylate) [70]. The perforated lamella in the previous reports had a core-shell-corona character and tetragonally ordered perforations, while in our case the sheets have a Janus character and pores are likely in a hexagonal order (Figure 6D). The fast Fourier transformation of larger areas gives a hexagonal scatter pattern and areas of overlapping sheets produce a pattern that is characteristic for structures with overlapping hexagonal features [71]. Above 50 wt% hPB, sheets with homogenous contrast are found, which agrees well with our previous results from bulk film analysis, as well as our simulations (Figure 6E).

## 4. Conclusions

We demonstrated that blending of B homopolymer into lamella morphologies of ABC triblock terpolymers is a promising route for tuning the B microphase and thus the shape of Janus nanostructures after crosslinking. Experiments and DPD simulations were in very good agreement regarding observed morphologies as well as morphological transitions. This combined approach helped to gain valuable insight into formation and stability of sandwiched microphases and offers guidelines to target specific morphologies for other ABC/B terpolymer blends. The formation of perforated Janus sheets is of particular interest, as BCPs have demonstrated to be a versatile and scalable source for isoporous membranes [72,73]. Due to the uniform porosity, BCP membranes have successfully been applied in water purification, as the nanometer-sized holes are suitable to withhold the smallest impurities [74,75]. Janus membranes have likewise generated considerable interest in recent years due to their application in selective separation [57]. The presented perforated nanosheets have pore sizes of about 20 nm, Janus character, and are flexible, which will allow studies on directional adsorption to interfaces and selective separation of nanoparticles or molecules depending on chemistry. We believe that the presented blending route will give access to a vast library of perforated Janus nanosheets with tuneable properties depending on the used ABC triblock terpolymer.

## Figures and Tables

**Table 1:** Amount of added hPB from 0%–100% relatively to the PB block of SBM and resulting composition of the SBM triblock terpolymer.

Code	hPB added ^a^	*f*_S_^b^	*f*_B_^b^	*f*_M_^b^	*Thickness PB ^c^*
**SBM-0**	0	40	22	38	10.9
**SBM-10**	10	39	24	37	15.0
**SBM-25**	25	38	26	36	12.8
**SBM-50**	50	36	30	34	12.6
**SBM-75**	75	34	33	33	12.9
**SBM-100**	100	33	36	31	14.6

^a^ amount of hPB added in (%) with respect to PB of SBM; ^b^ weight fractions of the respective polymer blend in [wt%]; ^c^ thickness in (nm) according to grey scale analysis (see SI).

**Table 2:** Interaction parameters for the S-M, S-B, and B-M particle pairs.

Interaction pairs	χ	χN	$\chi_{\mathrm{eff}}$	A
**S-M**	0.041	44.8	1.5	30.4
**S-B**	0.19	192	8.4	54.5
**B-M**	0.12	130	5.6	44.5

## Supplementary Materials

Supplementary Figures on alternative crosslinking and grey scale analysis are available online at https://www.mdpi.com/2073-4360/11/7/1107/s1.
