# Supplementary material for: Janus Nanostructures from ABC/B Triblock Terpolymer Blends
Source: Polymers (Basel). 2019 Jun 30;11(7):1107. doi: 10.3390/polym11071107 (PMC6680841; doi:10.3390/polym11071107)
Supplement: Supplementary file 1 [file polymers-11-01107-s001.pdf]

Supporting Information

to

Janus nanostructures from ABC/B triblock terpolymer blends

Andrea Steinhaus, Deepika Srivastva, Arash Nikoubashman, André H. Gröschel

Supporting Figures

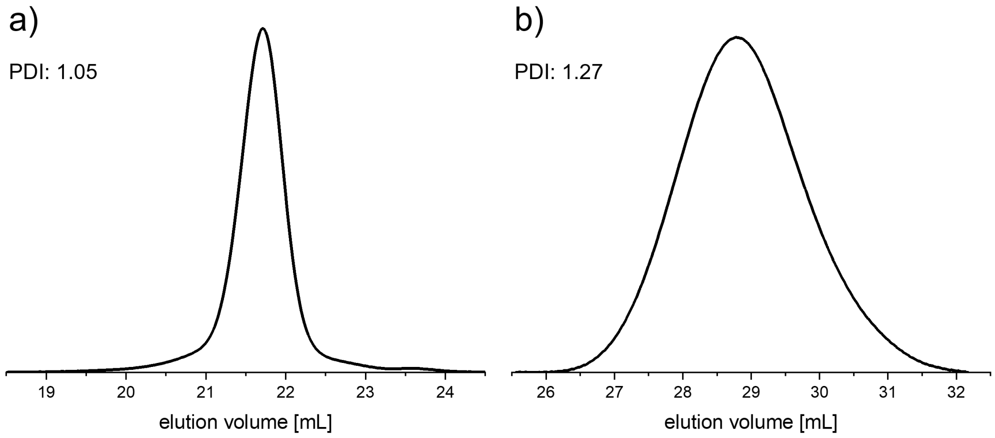

Figure S1: SEC elugrams of the used polymers. a) SBM triblock terpolymer and **b)** the polybutadiene homopolymer hPB.

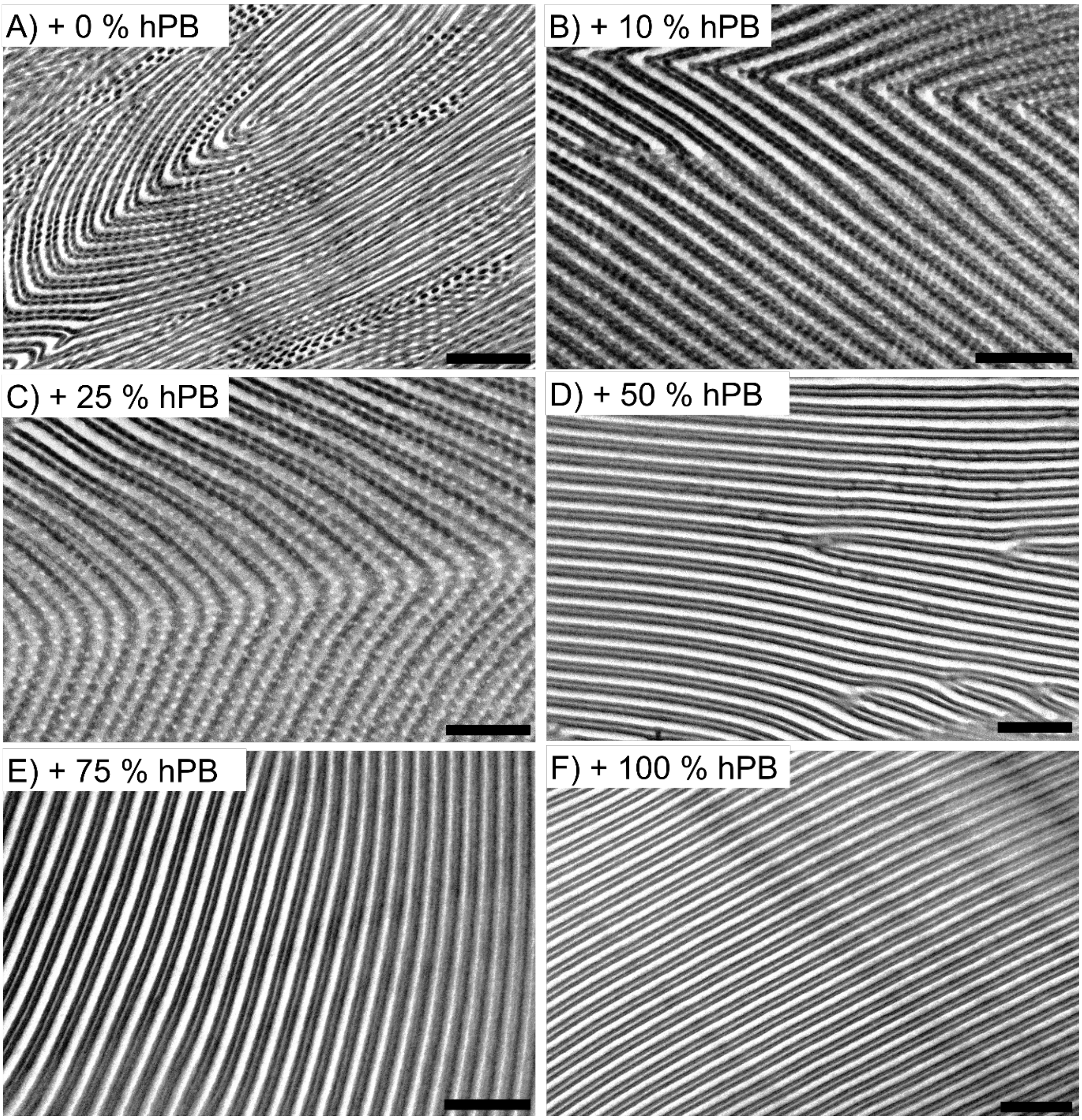

Figure S2: TEM overview images of morphologies cross-linked with S_2Cl_2. Added content of hPB indicated in the figure labels. Scale bar 200 nm.

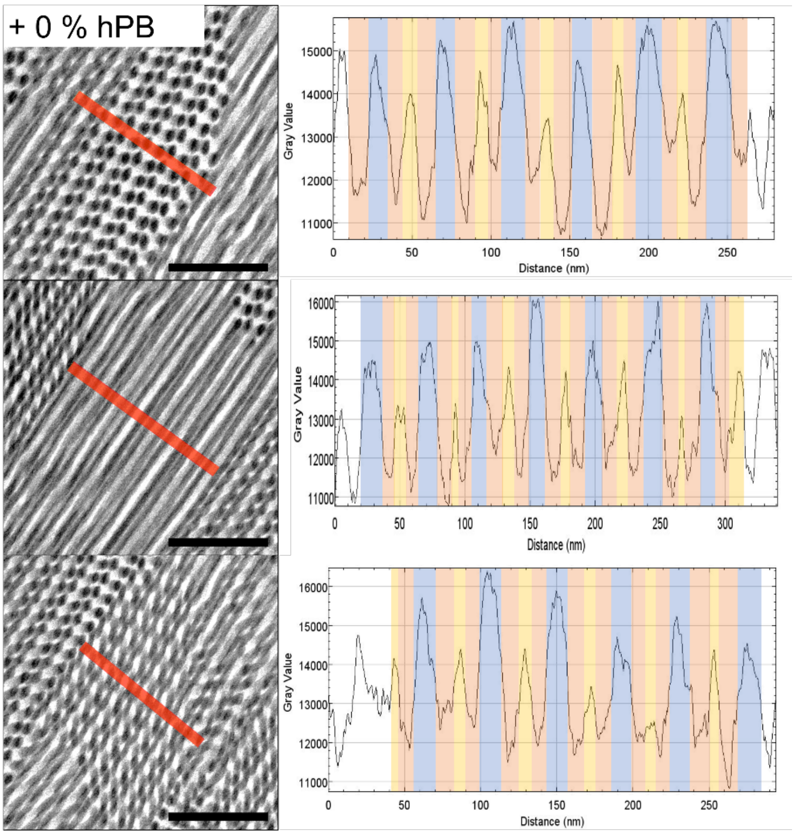

Figure S3: Grey scale analysis of selected locations of the bulk morphology with 0% hPB blending. Scale bar 200 nm.

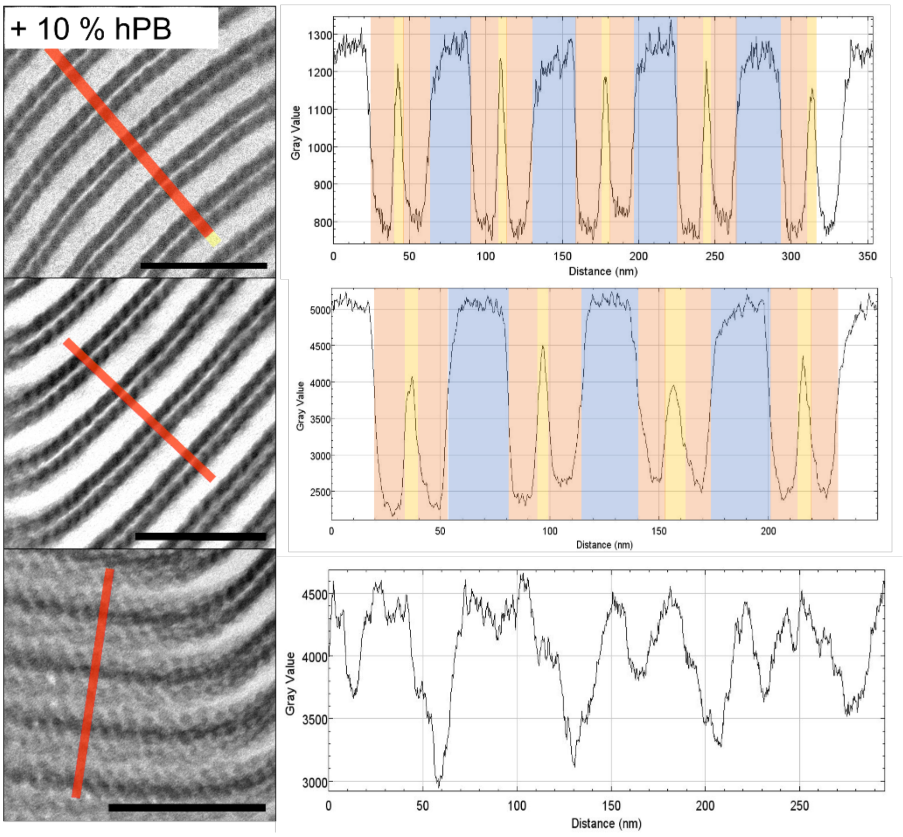

Figure S4: Grey scale analysis of selected locations of the bulk morphology with 10% hPB blending. Scale bar 200 nm.

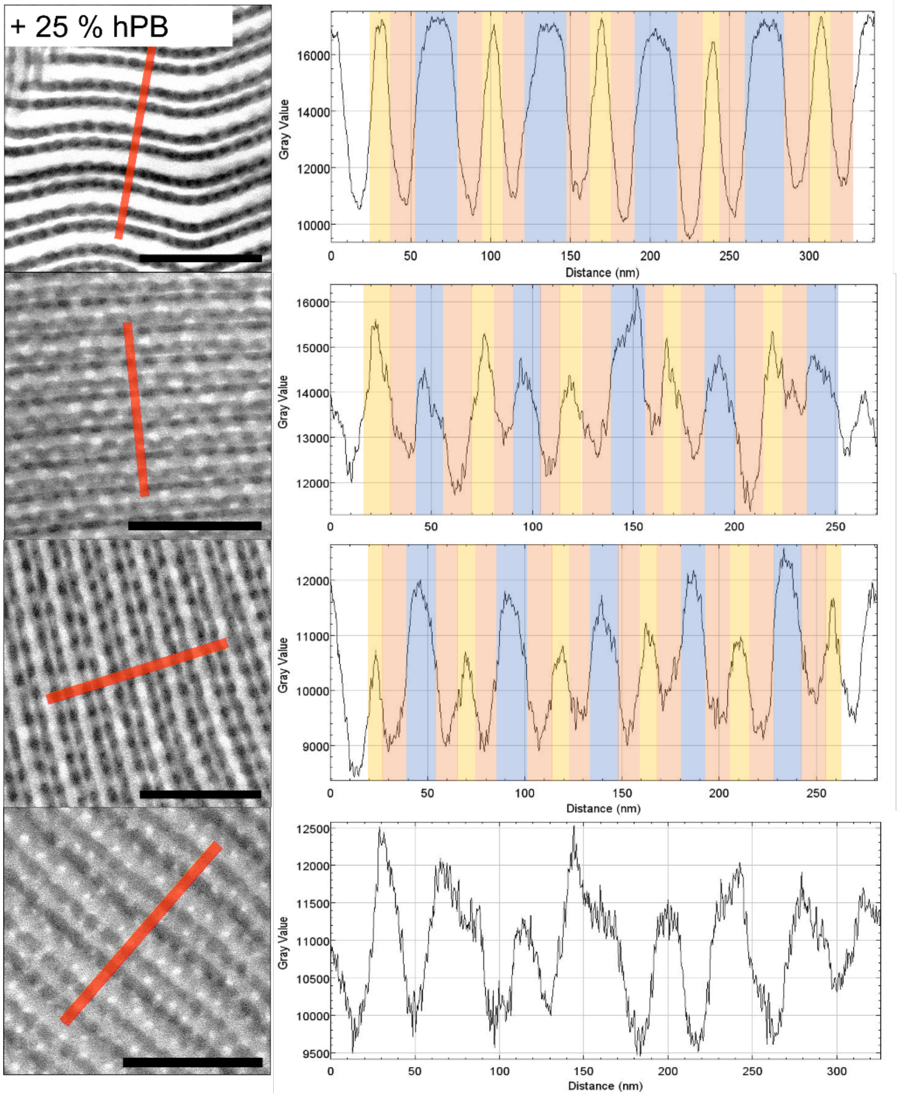

Figure S5: Grey scale analysis of selected locations of the bulk morphology with 25% hPB blending. Scale bar 200 nm.

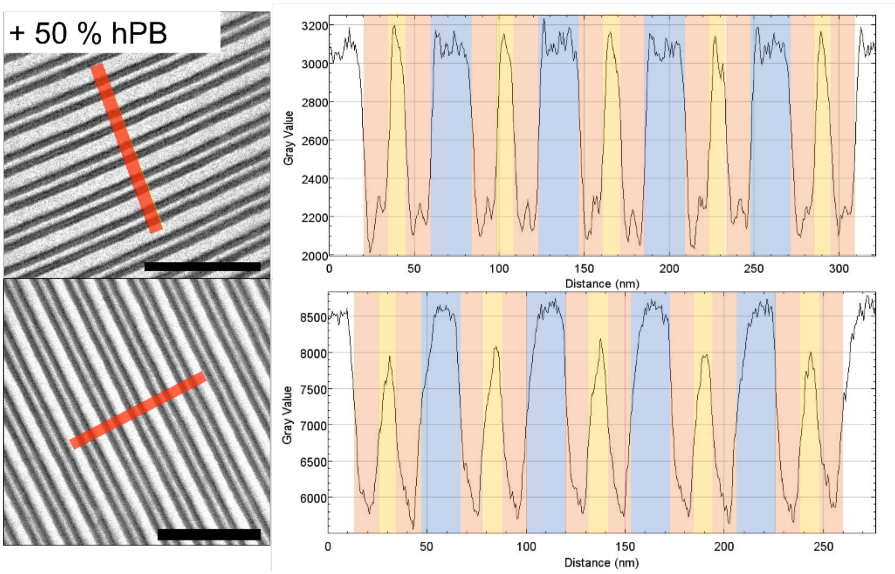

Figure S6: Grey scale analysis of selected locations of the bulk morphology with 50% hPB blending. Scale bar 200 nm.

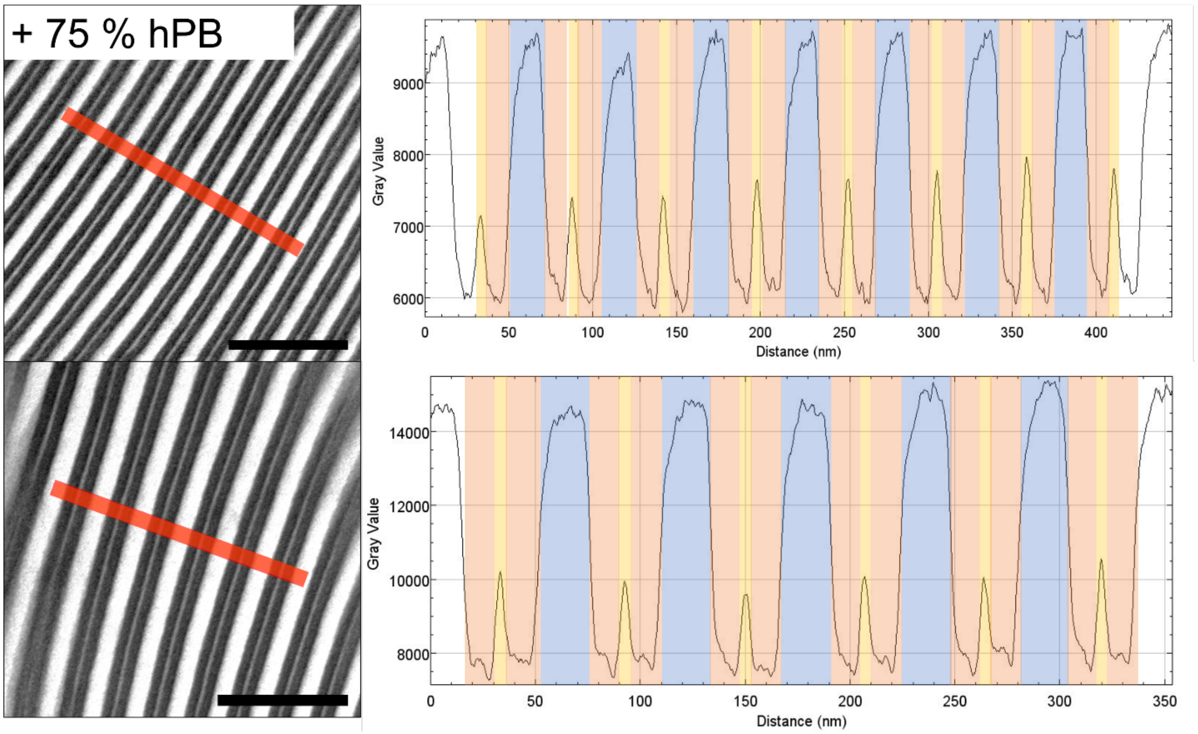

Figure S7: Grey scale analysis of selected locations of the bulk morphology with 75% hPB blending. Scale bar 200 nm.

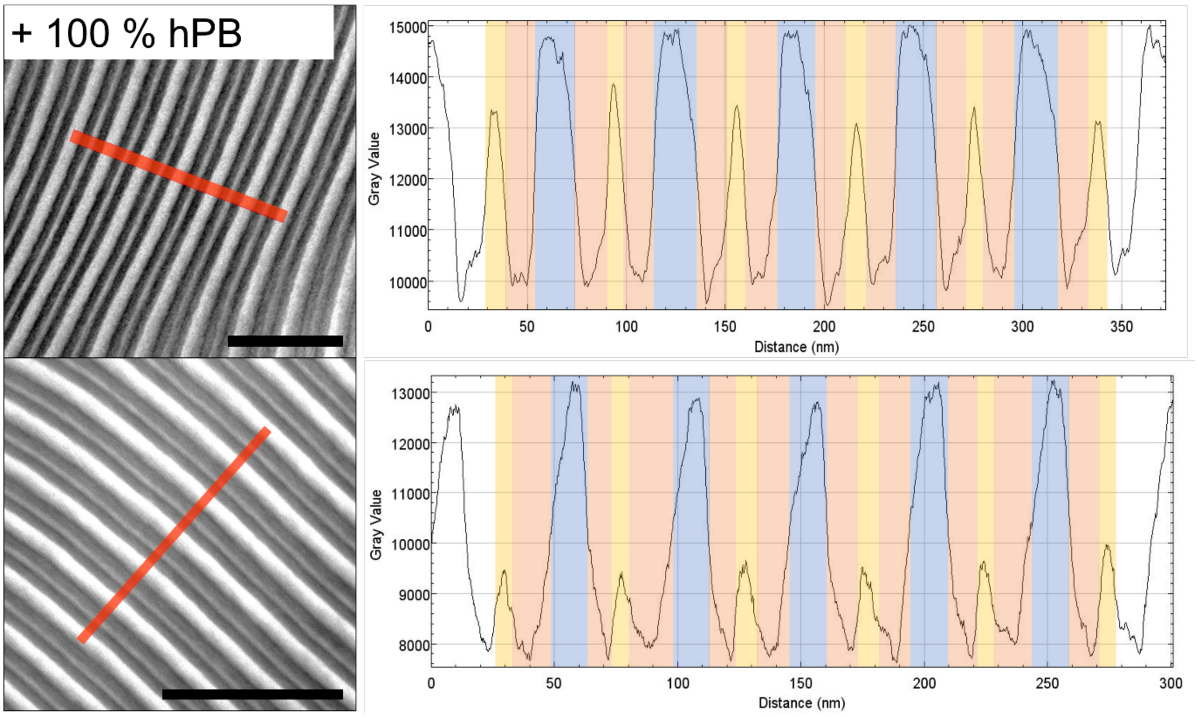

Figure S8: Grey scale analysis of selected locations of the bulk morphology with 100% hPB blending. Scale bar 200 nm.
